# Insulin-Related Peptide 5 is Involved in Regulating Embryo Development and Biochemical Composition in Pea Aphid with Wing Polyphenism

**DOI:** 10.3389/fphys.2016.00031

**Published:** 2016-02-09

**Authors:** Shan-Shan Guo, Meng Zhang, Tong-Xian Liu

**Affiliations:** State Key Laboratory of Crop Stress Biology for Arid Areas, Northwest A&F UniversityYangling, China

**Keywords:** aphid, insulin-like peptide, wing polyphenism, wing differentiation, biochemical components

## Abstract

In aphids there is a fecundity-dispersal trade-off between wingless and winged morphs. Recent research on the molecular mechanism of wing morphs associated with dispersal reveals that insulin receptors in the insulin signaling (IS) pathway regulate alternation of wing morphs in planthoppers. However, little is known about whether genes in the IS pathway are involved in developmental regulation in aphid nymphs with different wing morphs. In this study, we show that expression of the insulin-related peptide 5 gene (*Apirp5*) affects biochemical composition and embryo development of wingless pea aphids, *Acyrthosiphon pisum*. After comparing expression levels of major genes in the IS pathway between third instar winged and wingless nymphs, we found that *Apirp5* showed higher expression in head and thorax in the wingless nymphs than in the winged nymphs. Although microinjection treatment affects physical performance in aphids, nymphs with RNA interference of *Apirp5* had less weight, smaller embryos, and higher carbohydrate and protein contents compared to the control group. Comparison between winged and wingless nymphs showed a similar trend. These results indicate that *Apirp5* is involved in embryo development and metabolic regulation in wing dimorphic pea aphid.

## Introduction

Fecundity-dispersal trade-offs strongly drive the life-history evolution of insects with wing polyphenism. Because of their short lifespan, allocation of limited nutritional resources differs for insects that engage in optimal reproduction or long-distance migration. Active dispersal can decrease the fecundity of insects (Bonte et al., 2012), whereas reproduction may limit migration during a lifecycle. The trade-off between fecundity and dispersal is best studied in species with the trait of wing dimorphism, including pea aphid (*Acyrthosiphon pisum*; Braendle et al., 2006; Brisson, 2010), sand cricket (*Gryllus firmus*; Mole and Zera, 1993), and planthopper (*Prokelisia dolus*; Denno et al., 1989). Typically, the flight-capable morph (winged or long-wing) has fully developed wings and muscle apparatus and undeveloped ovaries, but in contrast, ovaries grow rapidly and early in the flightless morph (wingless or short-wing), and these differences can be identified during nymphal stages. The choice to reproduce or disperse is usually governed by environmental conditions and/or genetic backgrounds. The molecular mechanism governing the wing morph has been revealed for the migratory brown planthopper (*Nilaparvata lugens*); two insulin receptors in the insulin signaling (IS) pathway play a regulatory role in governing wing morph determination (Xu et al., 2015). However, very little is known about the molecular mechanism of developmental and physiological changes in nymphs with wing polyphenism.

Photoperiod could affect the reproductive mode of aphids. Sexual reproduction occurs when the days grow shorter in the late summer, and eggs are produced by mated female aphids for overwintering. Under long day and warm conditions, developing embryos can be found in the ovarioles of nymphs and adult parthenogenetic viviparous aphids. The aphid also shows wing polyphenism, in which asexual aphids with an identical genetic background can produce winged or wingless progeny under long day conditions (Sutherland, 1969). Aphids with different wing morphs show a fecundity-dispersal trade-off and distinct growth rate in the same tissues (Dixon et al., 1993; Ishikawa et al., 2008; Ishikawa and Miura, 2009). Newly molted wingless adult aphids have highly developed ovaries for rapid reproduction, and winged adult aphids possess indirect flight muscles and wings for long distance migration (Ishikawa and Miura, 2009). Wing dimorphism is evident during the aphid nymphal phase. However, during the first and second instar stages, it is impossible to distinguish winged nymphs from wingless nymphs by wing apparatus morphology (Ishikawa et al., 2008). In the third instar, nymphs destined to be winged aphids have visible wing primordia on mesothorax, whereas no wing primordia are present on wingless nymphs (Ishikawa et al., 2008; Ogawa and Miura, 2013). Embryos in the ovary of wingless nymphs develop faster than those of winged aphids during the entire nymphal stage (Dixon and Howard, 1986; Ishikawa and Miura, 2009). Such morphological and physiological differences clearly separate the development of winged aphids from wingless aphids (Braendle et al., 2006; Simpson et al., 2011).

The insulin and insulin growth factor signaling pathways play diverse roles in regulation of growth, metabolism, reproduction, behavior, and aging in invertebrates and vertebrates (Garofalo, 2002; Taniguchi et al., 2006; Antonova et al., 2012). The insulin signaling (IS) pathway is activated by insulin-like peptides (ILPs, also called insulin-related peptides, IRPs) through the insulin receptor (InR), which activates a sequence of downstream proteins, such as the insulin receptor substrate (IRS). Signaling in this pathway can be downregulated by the insulin degrading enzyme (IDE), which degrades insulin intracellularly (Shen et al., 2006). Evidence shows that the IS pathway regulates alternative reproductive phenotypes in insects by controlling resource allocation during tissue development (Emlen et al., 2006; Wheeler et al., 2006; de Azevedo and Hartfelder, 2008). *Amilp2* dsRNA treatment results in a reduction in ovary size in the honey bee (Wang et al., 2013), and ovariole number is decreased by *irs* dsRNA feeding (Wolschin et al., 2011). In another example, the *FOXO* gene, a downstream nexus in the IS pathway, directly regulates the size of copulatory organs in male dung beetle *Onthophagus nigriventris*, and is affected by nutritional polyphenism (Snell-Rood and Moczek, 2012).

The pea aphid *A. pisum* (Harris) is an important crop pest and a study model with wingless and winged morphs (Brisson and Stern, 2006; Figures S1A,B) that reflect a fecundity-dispersal trade-off. External environmental signals, including crowding, poor-quality diet, and predators, stimulate adult female aphids to produce nymphs that undergo wing differentiation and other related changes in organs and tissues (Sutherland, 1969; Brisson, 2010; Ogawa and Miura, 2013). How these signals are transduced internally in nymphs is not known. Based on the role of the IS pathway in regulating wing dimorphism in other insects, we first examined the conservation of ILPs and signaling proteins, as revealed in the pea aphid genome (Consortium, 2010). Previous studies identified 10 insulin-related peptide genes (*Apirp*s), two insulin receptor genes (*Apinr*s), one insulin receptor substrate gene (*Apirs*), and two insulin degrading enzyme genes (*Apide*s) in pea aphid (Consortium, 2010; Huybrechts et al., 2010; Antonova et al., 2012). The ApIRPs are divided into three groups based on cleavage sites and cysteine spacing (Huybrechts et al., 2010). The first group includes ApIRP1 to ApIRP4 with expected cysteine spacing and canonical prohormone convertase cleavage sites. The second group (ApIRP5, ApIRP6, and ApIRP7) is likely cleaved by furin. Notably, *Apirp5* transcripts are highly represented in EST sequences, suggestive of high expression and an important role in rapid development of aphids (Huybrechts et al., 2010). The third group, including ApIRP8 to ApIRP10, has a different cysteine spacing from the first group (Huybrechts et al., 2010). We hypothesized that genes in the IS pathway would show different expression patterns, and that some of the genes were involved in regulation of development and biochemical components in aphid nymphs with or without wing primordia. In this study, we focused on the third instar pea aphid, because it is the earliest phase when winged nymphs can be identified by visual inspection. After comparing the transcript levels of genes in the IS pathway between third instar winged and wingless aphids, we selected *Apirp5* for RNA interference (RNAi). Analysis of morphological and physiological changes of pea aphids after *Apirp5* RNAi treatment revealed functions of *Apirp5* on regulation of embryo development and biochemical components in wingless nymphs.

## Methods

### Pea aphids

*A. pisum* used in this study was collected from Yunnan Province, China, and maintained on potted broad bean (*Vicia faba* L.) under long-day (LD) conditions (14 h light: 10 h dark, 20 ± 2°C).

### Candidate genes in the insulin signaling pathway

Sequences of *Apirps, Apinrs, Apirs*, and *Apides* were obtained from Huybrechts et al. (2010) and from the National Center for Biotechnology Information (NCBI) via Blast searches of the pea aphid genome (www.ncbi.nlm.nih.gov/BLAST/) with homologous proteins sequences from *Drosophila melanogaster* and *A. mellifera*. The protein domains of predicted *Apinr, Apirs*, and *Apide* genes were analyzed and identified by searching the Self-Monitoring, Analysis and Reporting Technology database (http://smart.embl-heidelberg.de/). Multiple sequence alignments were performed by using MEGA 6.0 (Tamura et al., 2013).

### Experimental treatments

Winged aphids were induced under a high-density (HD) condition (30 wingless adult aphids per seedling) as described by Ishikawa et al. (2008) and Sutherland (1969). Thirty wingless adult aphids were caged on a broad bean seedling that was ~2 cm in height. After 24 h, adult aphids were removed, and the offspring were reared on the broad beans until they developed to third instar when winged nymphs show visible wing primordia (Ishikawa et al., 2008). The induction rate of winged aphids was 47.8 ± 5.3% (*n* = 836). In order to minimize the effects of development rate of winged and wingless nymphs on gene expression pattern and biochemical contents, we weighed and dissected third instar nymphs on the fifth day after birth because each of the first three instars in both winged and wingless aphids lasted for 2 days, and winged and wingless nymphs could not be visually identified until the third instar. Five days after removing adult aphids, all third instar nymphs were individually weighed, and 30–40 third instar aphids with or without wing primordia were dissected in 0.1 M phosphate-buffered saline (PBS, pH 7.4). The antenna, head without antenna, thorax, midgut, and abdomen without midgut were separately collected. For the treatments above, each had four independent replicates. To avoid the known effects of HD conditions on promoting the wing polyphenism (MacKay and Wellington, 1976), female asexual aphids were reared at low density (LD; three aphids per seedling) for longer than three generations before experiments.

### RNA extraction and cDNA synthesis

Total RNA was extracted using RNAiso Plus reagent (TaKaRa, Dalian, China) following the manufacturer's protocol. The RNA was quantified using a NanoDrop® 2000c (Thermo Fisher Scientific, Middletown, VA, USA) at 260 nm. To generate the first strand cDNA, 1 μg of total RNA was reverse-transcribed in 20 μl volume using the PrimeScript RT reagent Kit With gDNA Eraser (TaKaRa, Dalian, China).

### Real-time quantitative PCR

**Figure 1:**
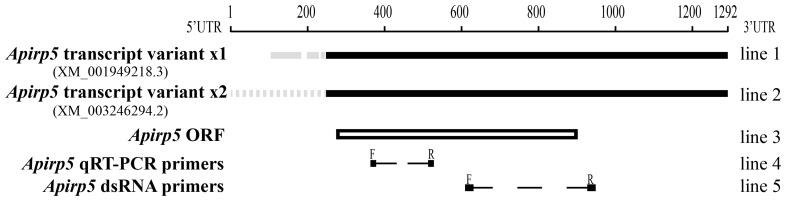
**Comparison of ***Apirp5*** mRNA sequences**. Two transcript variants of *Apirp5* (line 1 and line 2) share the same predicted ORF (box of line 3), and there is no overlap between the products of *Apirp5* qRT-PCR and *Apirp5* dsRNA (line 4 and line 5). In line 1 and line 2, the black lines show the same sequences in the two transcript variants, and gray solid line and gray dotted line show different sequences in the 5′UTR. In lines 4 and 5, dotted lines show the products of *Apirp5* qRT-PCR and dsRNA, and short lines are primers of two products.

The reactions were performed with iQ5 real-time cycler (Bio-Rad, Hercules, CA, USA) and SYBR® Premix Ex Taq™ II (TaKaRa, Dalian, China). The primers to each target transcript were listed in Table 1. The qRT-PCR amplified fragment of *Apirp5* did not overlap with *Apirp5* dsRNA (Figure 1). Running parameters were 94°C for 5 min, followed by 40 cycles at 94°C for 30 s, 57°C for 30 s, and 72°C for 1 min. Considering the high homology present in several insulin genes, the real-time PCR products were extracted from 1% agarose gel and purified using a gel extraction kit (Axygen Scientific, Union City, CA, USA) for cloning into pGEM-T Easy Vector System (Promega, Madison, WI, USA) and submitted for sequencing (Life technology Inc., Shanghai, China). To ensure that only a single product was amplified, we carried out a melting curve analysis for each qRT-PCR reaction. Mean PCR efficiency values for each gene were calculated from individual amplification by using LinRegPCR software (Ramakers et al., 2003), which also provided the baseline and cycle threshold (ct). Relative gene expression data were analyzed following the Pfaffl method (Pfaffl, 2001) with normalization to the *A. pisum* ribosomal protein (Rp) L7 transcript (Nakabachi et al., 2005). Non-expression was defined as a relative quantity lower than 0.0001.

**Table 1:** **List of accession numbers for genes encoding insulin signal components in the pea aphid, and primers for real-time quantitative PCR and dsRNA synthesis**.

**Gene**	**Peptide**	**mRNA ID**	**Protein ID**	**Primers**	**Size**
*Apirp1*	ApIRP1	XM_003247500.2	XP_003247548.1	F 5′ CACTTAACAGTTTACCACCTTTCAAC 3′	154
				R 5′ TGTTGAGGTGGTAGTTTCAAATCG 3′	
*Apirp2*	ApIRP2	XM_003244078.2	XP_003244126.1	F 5′ CTCTATGCAAAAGCAACTACAATAGC 3′	272
				R 5′ GCGGCACACAATTAAGTCTACTAT 3′	
*Apirp3*	ApIRP3	XM_003240882.2	XP_003240930.1	F 5′ CGTGGCAGTAGTACGAATATAC 3′	91
				R 5′ GCCGTCACTAAATAGATATATTATG 3′	
*Apirp4*	ApIRP4	XM_001949403.3	XP_001949438.1	F 5′ GAGCAAGGAGCTGAAA 3′	81
				R 5′ CAACTCGGTACAAGACG 3′	
*Apirp5x1*	ApIRP5	XM_001949218.3	XP_001949253.1	F 5′ GAGGCATTTCTGTGGA 3′	166
*Apirp5x2*	ApIRP5	XM_003246294.2	XP_003246342.1	R 5′ TTTCAGGTGATGTGGC 3′	
*Apirp6*	ApIRP6	XM_003240685.2	XP_003240733.1	F 5′ GGGCTCCTGAAGTTATAGAATGGC 3′	415
				R 5′ TTTTGTTTGAAATAATCGGTACAGC 3′	
*Apirp7*	ApIRP7		ACYPIG753272	F 5′ AACGAGTTACCTGCTGGAGAGG 3′	144
				R 5′ ATACTTTAGGCATCCGGAGCAC 3′	
*Apirp8*	ApIRP8	XM_008187642.1	XP_008185864.1	F 5′ GATGGTCCTTACTGGAACGAAAG 3′	221
				R 5′ AGATATAATGCTTCCGGTTCAGG 3′	
*Apirp9*	ApIRP9		ACYPIG831367	F 5′ TGATTGTGATTTTGTTGGTTGTGAG 3′	206
				R 5′ ATTGTGCAACAATCGTCCACG 3′	
*Apirp10*	ApIRP10		ACYPIG687176	F 5′ TCCTTTGTTATTTTATTGGCTCTGC 3′	82
				R 5′ CGGTATCCCAGTACAGATCATAACC 3′	
*Apinr1*	ApInR1	XM_008184754.1	XP_001942660.2	F 5′ CCAAGACCCACCCACCACT 3′	158
		XM_001942625.3	XP_008182977.1	R 5′ TAGGAACGCCATACCATCAGC 3′	
		XM_008184755.1			
*Apinr2*	ApInR2	XM_008187695.1	XP_008185917.1	F 5′ TGCCTCCAATAGTCGCACAAC 3′	138
				R 5′ CCAACATCTCAATAACTTCCCAAGG 3′	
*Apirs*	ApIRS	XM_003242381.2	XP_003242430.1	F 5′ CACCTCCAACATCTTCACATACG 3′	173
		XM_008181751.1	XP_003242429.1	R 5′ TACTACTTGCTGCTTCCCACACA 3′	
		XM_003242382.2			
		XM_008181752.1			
*Apide1*	ApIDE1	XM_001944696.3	XP_001944731.2	F 5′ TCATTAGCGACCATGAACGTCC 3′	182
				R 5′ AAAATTCGTTTCTGCGTCGTCC 3′	
*Apide2*	ApIDE2	XM_001942853.3	XP_001942888.2	F 5′ AGCTGCTATTGATGACGTTAAGATTG 3′	214
		XM_008182127.1	XP_008180349.1	R 5′ GATCACTTGTTGTTTCGAATAATGC 3′	
*Rpl7*	Rpl7	NM_001135898.1	NP_001129370.1	F 5′ GCGCGCCGAGGCTTAT 3′	81
				R 5′ CCGGATTTCTTTGCATTTCTTG 3′	
dsApirp5				F T7promoter+CAAAAACGTGAAACCCCAGAAA^*^	343
				R T7promoter+TCAAAAGTGGAAGACGAGAGCAG^*^	
dsgfp				F T7promoter+GCGACGTAAACGGCACA^*^	613
				R T7promoter+CGAACTCCAGCAGGACCAT^*^	

* *Sequence of T7 promoter: TAATACGACTCACTATAGG*.

### Double stranded RNA (dsRNA) synthesis and delivery

Specific primers designed for dsApirp5 were used to amplify target sequence from cDNA derived from adult aphid heads (Table 1). Cycling conditions were 95°C for 5 min, followed by 30 cycles at 95°C for 30 s, 55°C for 30 s, 72°C for 30 s, and then 72°C for 5 min. The PCR product was extracted from 1% agarose gel and purified using a gel extraction kit (Axygen Scientific, Union City, CA, USA). The product was cloned (pGEM-T Easy Vector System, Promega, Madison, WI, USA) and submitted for sequencing (Life Technology Inc., Shanghai, China). To synthesize dsApirp5, two separate PCR reactions were performed using a gene specific primer and a primer with terminal 5′ T7 promoter site (Table 1). Amplification strategies were 95°C for 1 min, followed by initial 10 cycles at 95°C for 30 s, 55°C for 30 s, 72°C for 30 s, and then 25 cycles at 95°C for 30 s, 60°C for 30 s, 72°C for 30 s, and then 72°C for 5 min. Purified PCR products were used as templates for dsRNA synthesis using the T7 RiboMAX™ Express RNAi System (Promega, Madison, WI, USA). The control dsRNA was prepared using *gfp* gene with primers listed in Table 1.

About 80–100 second instar pea aphids were maintained under a LD condition for 24 h, and dsRNA (200 nl, 8 mg/ml) was injected into each aphid with an MM33 links microinjector (Märzhäuser, Wetzlar, Germany). The needles were prepared by a P-97 Flaming/Brown Micropipette Puller (Sutter Co, Novato, CA, USA) using Borosilicate glass capillaries. The precise injection volume was controlled by an MM33 micromanipulator (Märzhäuser, Wetzlar, Germany). Each injected aphid was reared on a leaf disc kept on a thin layer water-agar gel (0.9% agar) in a 24-well plate. Two days later, aphids molting into third instar nymphs were individually weighed using Cubis® Ultramicro Balance with 0.001 mg readability (Sartorius, Göttingen, Germany), and then 22 treated and 22 control aphids were frozen in liquid nitrogen, and kept at –80°C before use, and 15 treated and 15 control aphids were dissected. The treatments were repeated four times. The aphids that died during experiments were removed.

### Size of embryos

The embryo size was measured from four groups of third instar nymphs: aphids with or without wing primordia produced under the HD condition, and aphids injected with dsApirp5 or dsgfp. The aphids were dissected under a dissecting microscope, and the embryos surrounded by a very thin layer of ovaries were photographed through an EVOS FL microscope (Life Technology Inc., Gaithersburg, MD, USA). The length of the five largest embryos in the ovary of each aphid was measured using ImageJ and analyzed (Abràmoff et al., 2004; Ishikawa and Miura, 2009).

### Protein, glycogen, and soluble carbohydrate assay

Protein, glycogen, and soluble carbohydrate were measured from third instar winged and wingless nymphs reared under HD condition, and also from third instar nymphs subjected to RNAi (dsgfp injected and dsApirp5 injected).

Eleven third instar nymphs with or without wing primordia were randomly collected from plants under HD inducing conditions for metabolic analysis. Third instar nymphs from dsRNA treatments were collected. Each whole aphid was measured individually as reported (Foray et al., 2012). After homogenizing in 180 μl lysis buffer solution (100 mM KH_2_PO_4_, 1 mM dithiothreitol, and 1 mM ethylenediaminetetraacetic acid, pH 7.4) at room temperature, 50 μl was removed to a new 1.5 ml microtube for protein assay following the method of Bradford (Kruger, 2009). The protein content was measured using a NanoDrop® 2000c (Thermo Fisher Scientific, Middletown, VA, USA) at 595 nm with bovine serum albumin as the standard. The rest was added with 37.5 μl of 20% Na_2_SO_4_, 112.5 μl of lysis buffer solution, 300 μl of chloroform, and 600 μl of methanol. After vigorous vortexing, the sample was centrifuged at 180 g for 15 min at 4°C to remove glycogen from the supernatant. One hundred and fifty microliters of supernatant were transferred into a new microtube for soluble carbohydrate analyses. The pellet was kept for glycogen content determination. All glycogen and soluble carbohydrate contents were determined by the anthrone method (Van Handel, 1965) and the absorbance was measured at 625 nm with D-glucose as the standard. The treatment was repeated three times.

### Statistical analyses

Two-way ANOVAs were used to determine whether the levels of gene expression were significantly different across the five tissues or parts of aphid, and whether this variation was affected by wing morph of nymphs. Then, multiple comparisons were corrected by Sidak test. Prior to comparing other values using Student's *t*-test, homogeneity of variance and normality assumptions (Shapiro-Wilk normality test) of the raw data were tested. Significance was ascribed at *p* < 0.05. All analyses were performed, and the data were graphed in GraphPad Prism (GraphPad Prism version 6.01 for Windows, GraphPad Software, San Diego, CA, USA). No statistical methods were used to predetermine sample size. Figures and schematic illustrations were prepared using Adobe Illustrator CS5 (Adobe Systems, San Jose, CA, USA).

## Results

### Comparison of sequence features for selected genes in the is pathway

By comparing the products of real-time PCR with the NCBI database, we confirmed that the fragments amplified for all selected genes of *A. pisum* clone in this study were the same as sequences retrieved from pea aphid genome. In all retrieved *Apirp*s, only *Apirp5* was expressed in two transcript variants (*Apirp5* transcript variant x1 and x2) which shared the same ORF (open reading frame; sequence information of *Apirp5* is shown in Figure 1). Accession numbers of *Apirp7, 9*, and *10* mRNAs were not retrieved from NCBI, so the predicted mRNA sequences were spliced based on the pea aphid genome and their amino acid sequences (Table 1).

Two *Apinr* sequences were retrieved from the genome of *A. pisum* (Table 1), and one ApIRS and two ApIDEs were retrieved based on the amino acid sequences of *D. melanogaster* (Table 1). *Apinr1, Apirs*, and *Apide2* were transcribed to three, four and two transcript variants, respectively, and then were translated into different protein isoforms (Table 1 and Figure S5).

The functional domains of deduced ApInRs, ApIRS, and ApIDEs of *A. pisum* were aligned with those of other model insects (Figures S2–S4).

### Expression profiles of genes in the is pathway in third instar nymphs with different wing morphs

The third instar is the earliest phase in which winged nymphs could be identified, so the expressions of insulin related genes in the IS pathway were compared between the third instar nymphs with different wing morphs. Aside from the signal of amplification of *Apirp2, 3, 8, 9*, and *10*, other transcripts were detected.

**Figure 2:**
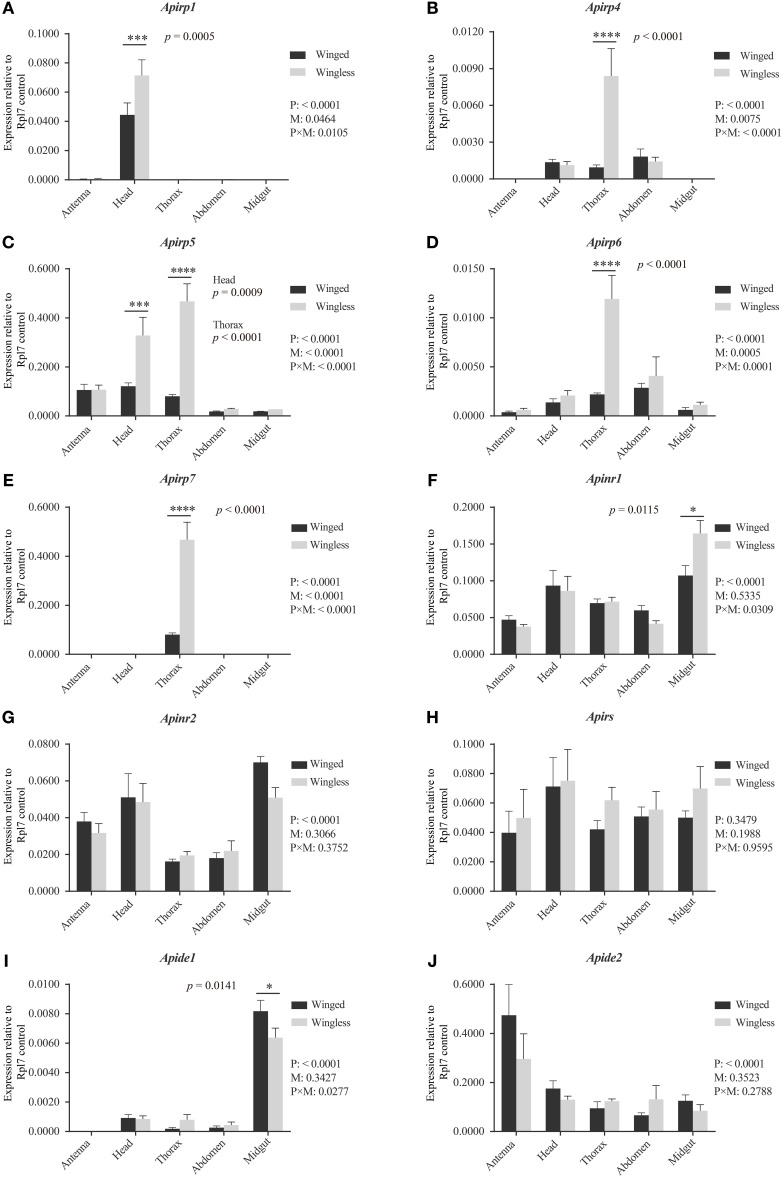
**Expression levels of ***Apirps, Apinr***s, ***Apirs***, and ***Apide***s in different tissues of winged (black column) and wingless (gray column) third instar pea aphid**. The abundance of *Apirps* **(A–E)**, *Apinrs* **(F,G)**, *Apirs* **(H)**, *Apides* **(I,J)** transcripts were compared among different tissues and between winged and wingless third instar aphids. Numbers under key are *P*-values resulting from two-way ANOVAs analyzing the effects of body parts (P), wing morphs (M), and parts × wing morphs (P × M). If the *P*-value of *P* is < 0.05, it means variation of target gene expression levels was affected by wing morph type. If the *P*-value of *M* is < 0.05, it means that the expression level of target gene show significant difference between two wing morphs considering all body parts. If the *P*-value of P × M is < 0.05, it means that the body parts and two morphs significantly affected difference comparison between the two wing morphs in each body part. Means and standard errors are shown from four independent biological replicates. Relative gene expression data were normalized to *Rpl7*, and multiple comparisons were corrected using Sidak test: ^*^p < 0.05; ^***^p < 0.001; ^****^p < 0.0001.

All expression differences of *Apirp*s between the two wing-morph nymphs showed the same trend: transcript expression was body-part specific and higher in wingless nymphs than in winged nymphs (Figures 2A–E). The expression of *Apirp1* was identified in antenna and head, although there was a significant difference in the heads between the winged and the wingless nymphs (Figure 2A). The transcripts of *Apirp4* existed in head, thorax and abdomen of the two wing-morph nymphs, and the expression level of *Apirp4* in the thorax of the wingless nymphs increased significantly compared with that of the winged nymphs (Figure 2B). The expression of *Apirp5* was detected in all tissues (Figure 2C). However, *Apirp5* transcript levels were significantly higher in wingless vs. winged nymphs in both head and thorax (Figure 2C). The transcripts of *Apirp6* were also detected in all tissues, and *Apirp7* existed only in the thorax (Figures 2D,E). The expression levels of *Apirp6* and *Apirp7* also showed significant increase in the thorax of the wingless nymphs (Figures 2D,E). *Apinrs, Apirs*, and *Apide2* transcripts were detected in all body parts (Figures 2F–H,J), but a higher level of *Apide1* transcription was evident in midgut of winged nymphs (Figure 2I).

### Body weight, ovaries or embryos, and nutrient content of wingless and winged nymphs, *Apirp5* RNAi wingless nymphs and controls

**Figure 3:**
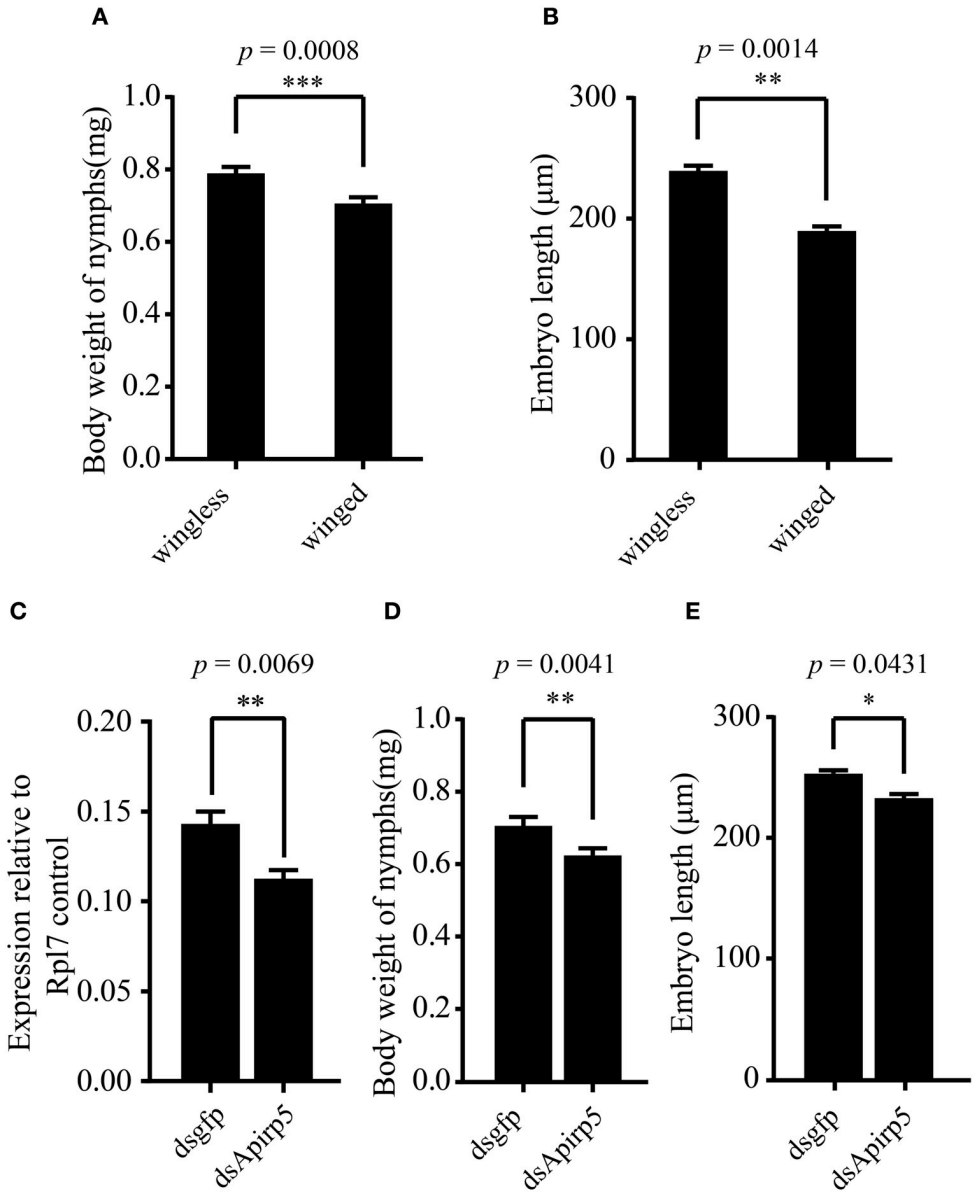
**Comparison of body weights and embryo lengths among different groups of third instar pea aphid nymphs and expression levels of ***Apirp5*** between wingless nymphs treated with dsgfp and dsApirp5. (A)** Body weights of wingless nymphs is greater than that of winged nymphs (*n* = 70 wingless, 92 winged), as is embryo lengths **(B)** (*n* = 14 wingless, 14 winged). **(C)** *Apirp5* expression is reduced in dsApirp5 treated wingless nymphs relative to that of dsgfp treated nymphs from four independent biological repeats. Relative gene expression data were normalized to *Rpl7*. **(D)** Body weight is reduced in dsApirp5 treated wingless nymphs (*n* = 32 dsgfp, 32 dsApirp5), as is embryo lengths **(E)** (*n* = 25 dsgfp, 29 dsApirp5), relative to that of dsgfp treated nymphs. Means and standard errors are shown. Data analyzed with Student's *t*-tests: ^*^p < 0.05; ^**^p < 0.01; ^***^p < 0.001.

**Figure 4:**
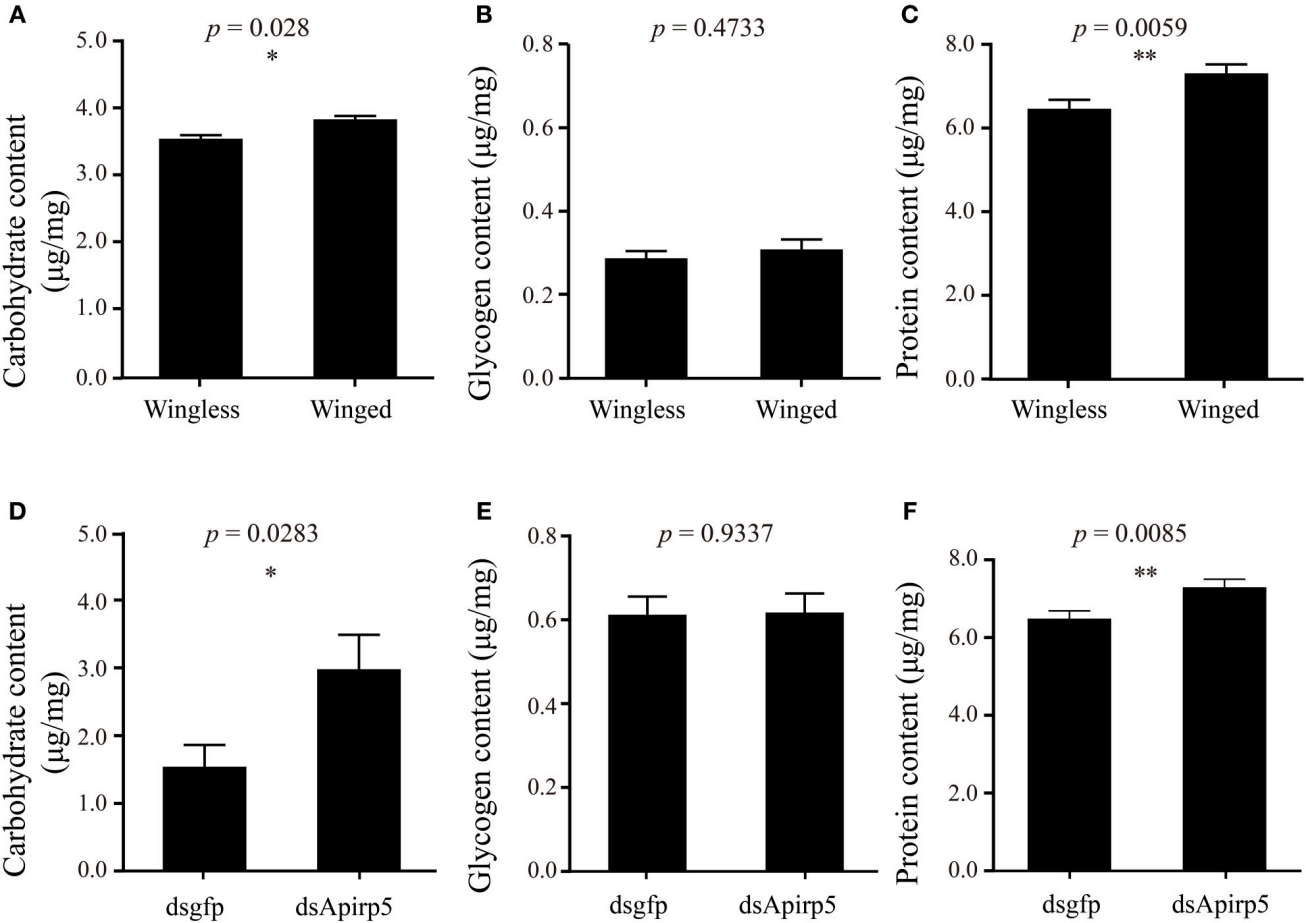
**Contents of carbohydrate (A,D), glycogen (B,E) and protein (C,F) in third instar nymphs**. The comparison of carbohydrate **(A)** (*n* = 31 wingless, 28 winged), glycogen **(B)** (*n* = 32 wingless, 32 winged) and protein **(C)** (*n* = 32 wingless, 32 winged) contents between winged and wingless nymphs is shown in the top row. In the second row, the carbohydrate **(D)** (*n* = 12 dsgfp, 13 dsApirp5), glycogen **(E)** (*n* = 42 dsgfp, 28 dsApirp5) and proteins **(F)** (*n* = 29 dsgfp, 42 dsApirp5) are compared between dsgfp and dsApirp5 treatments in wingless nymphs. Means and standard errors are shown. Data analyzed with Student's *t*-tests: ^*^p < 0.05; ^**^p < 0.01.

Differences in the phenotype of insects is associated with differentiation of organs or tissues and allocation of nutrient content. Third instar nymphs with wing primordia weighed less than wingless nymphs (Figure 3A). Embryos in the ovary of the wingless nymphs were significantly larger than those of the winged nymphs (Figure 3B and Figures S6A,B). Carbohydrate and protein contents of winged nymphs were significantly higher than those of wingless nymphs (Figures 4A,C), whereas glycogen content was similar between the wingless and winged nymphs (Figure 4B).

According to previous reports, the large majority of *Apirp5* ESTs in aphid libraries is likely involved in stimulating fast growth (Huybrechts et al., 2010). And in both of head and thorax, *Apirp5* showed differential expression between third instar nymphs with and without wing primordia (Figure 2C), so the functions of *Apirp5* on phenotypic plasticity of wing dimorphism were tested. Three days after dsApirp5 was injected into hemolymph of the second instar nymphs, expression levels of *Apirp5* were reduced significantly compared with dsgfp controls (Figure 3C). Compared with dsgfp control, the dsApirp5 group showed lighter body weight and smaller embryo size (Figures 3D,E and Figures S6C,D). However, the formation of wing structures was not observed in dsApirp5-treated and control nymphs.

Wingless nymphs treated with dsApirp5 also had significantly higher carbohydrate and protein contents than the dsgfp control nymphs (Figures 4D,F), but glycogen content was similar between the two group nymphs (Figure 4E).

## Discussion

**Figure 5:**
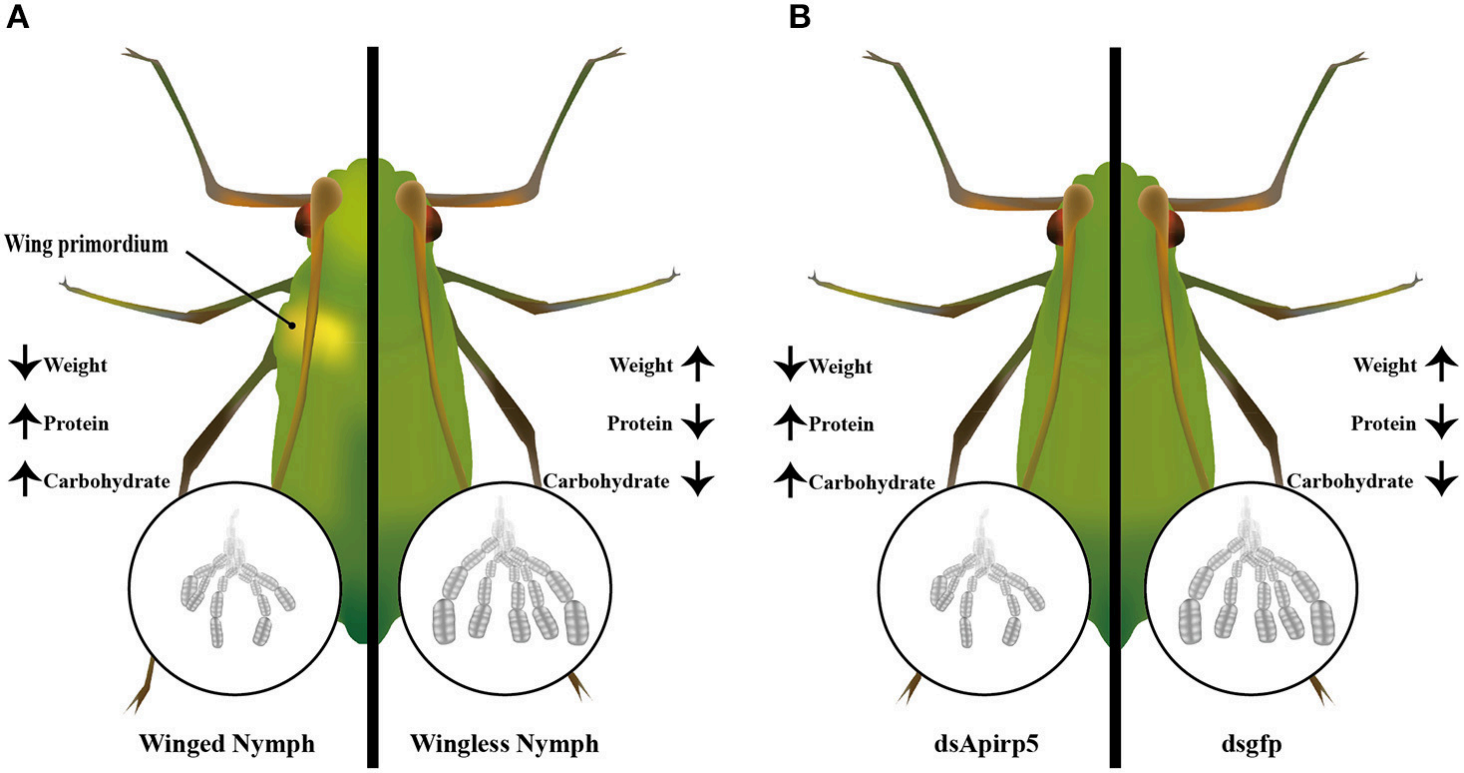
**Summary diagram of embryo development and biochemical components between the winged and wingless third instar nymphs (A), and dsApirp5 and dsgfp treated third instar nymphs (B)**. The knockdown experiment showed that *Apirp5* was involved in embryo development and regulation of carbohydrate and protein in wingless nymphs.

The IS pathway directly or/and indirectly affects polyphenism in insects (Wolschin et al., 2011; Snell-Rood and Moczek, 2012; Wang et al., 2013; Xu et al., 2015). For wing dimorphic insects, nymphs with or without wing primoridia often show different development status and physiological performance. In this study, combined with previous reports, we presented the expression of selected genes in the IS pathway, and identified one strong candidate gene—*Apirp5* (Figure 5). This report is the first study linking wing polyphenism with the IS pathway in aphids. Our results indicated that the IS pathway might have effects of regulation on embryo development and certain biochemical substance during wing differentiation in nymphs with wing polyphenism.

Of the 10 *Apirp*s identified, *Apirp2, 3, 8, 9, and 10* were not detected in the third instar nymphs. These undetected genes were probably expressed at low-levels in the third instar pea aphids or in special tissues or cells. The expression profiles of the other five *Apirp*s varied dramatically among different body parts, indicating that the expression of *Apirp*s might be associated with specific tissues. For instance, *Apirp1* was only detected in aphid's head (Figure 2A), which contains median neurosecretory cell (MNC) that secretes many important ILPs in other insects, whereas *Apirp7* transcript was only found in thorax (Figure 2E). However, *Apirp5* and *6* transcripts were detected in whole body (Figures 2C,D), implying that *Apirp5* and *Apirp6* might serve universal roles in the third instar nymphs of *A. pisum*. In the brown planthopper (*N. lugens*), *Nilp1* and *Nilp3* are highly expressed in head, fat bodies, legs, wing buds, and cuticle in the fourth instar nymphs (Xu et al., 2015). Therefore, different *irp* or *ilp* genes could be expressed in the same or different tissues of these hemipteran insects. Similar findings are reported for *Drosophila* (Ikeya et al., 2002; Broughton et al., 2005; Veenstra and Sellami, 2008) and *Aedes aegypti* (Riehle et al., 2006). Although the MNC in the medial region of brain is the main source of *irp* or *ilp* expression in many insects, the fat body is another important source (Antonova et al., 2012). Unlike winged nymphs, there are no muscle tissues inside the thorax of wingless nymphs. Fat bodies filling in the thorax cavity may play a role in increasing expression levels of *Apirp4, 5, 6*, and *7*. Because the IS pathway has been shown to affect juvenile hormone (JH) synthesis in other insects (Tatar et al., 2001; Tu et al., 2005), JH can inhibit wing development in aphids (Ishikawa et al., 2013). The higher expression levels of *Apirp*s in thorax of wingless nymph than winged nymph might promote higher JH in wingless nymphs. It seemed that the decrease in expression levels of *Apirp*s was associated with the winged phenotype of *A. pisum*.

Based on gene expression pattern, we found *Apirp5* showed significant expression differences between two wing-morph nymphs in both head and thorax (Figure 2C), which are the core areas for secreting hormone that is associating with polyphenism in some insects (Hartfelder and Emlen, 2012). Besides *Apirp5* and *7*, expression levels of the other *Apirp*s with significant differences between two morphs were low (Figures 2A,B,D,E). Because two pesudogenes are very similar to *Apirp7* (Huybrechts et al., 2010), transcripts from pesudogenes probably affected quantification of transcripts of *Apirp7*. Besides, due to previous reports that *Apirp5* with high expression abundance is likely a serious candidate for rapid growth in many aphids (Huybrechts et al., 2010), *Apirp5* was chosen to knockdown by RNA interference.

High reproduction rate has previously been observed in wingless aphids (van Emden and Harrington, 2007). Our results showed that the embryos in the ovaries of wingless nymphs were larger than those of winged nymphs, and similar results are also found in other aphid species (Ishikawa and Miura, 2009). Meanwhile we found that the bodies of the third instar wingless nymphs were heavier than the third instar winged nymphs. This was consistent with previous findings that wingless aphids have a shorter development time than winged aphids (Dixon and Howard, 1986), and winged aphids are smaller than wingless aphids (Zera and Denno, 1997). The results of RNAi experiments suggested *Apirp5* played a pivotal role in body weight and embryo development of *A. pisum*. The reduction of *Apirp5* transcripts resulted in weight loss and smaller embryo development of wingless nymphs, which were features of winged nymphs. In *A. mellifera*, ovariole numbers connected with queen and worker development decrease when *Amilp2* RNAi is conducted (Wang et al., 2013). In *Drosophila*, inhibiting expression of *dilp2, 3*, and *5* also causes development delay and serious weight loss (Rulifson et al., 2002). For another dipteran insect, *A. aegypti, Aaegilp3* is shown to control egg production (Brown et al., 2008). Therefore, influences of *Apirp5* on weights and embryo size of pea aphid indicated that this gene was likely involved in regulating embryo development during wing differentiation.

Further, we also found that the carbohydrate and protein contents were higher in winged nymphs than those in wingless nymphs. Such differences in nutritional metabolism between the two wing-morph nymphs could also be the result of IS pathway regulation and might be associated with their different morphological and physiological attributes. For instance, winged aphids possess indirect flight muscle and need fuel to provide energy for flight (Zera and Denno, 1997; Ishikawa et al., 2008). Therefore, high abundances of protein and trehalose, the main component of soluble carbohydrate in haemolymph (Wyatt and Kalf, 1957), are essential for flight muscle development and flight, respectively (Beenakkers et al., 1984; Marden, 2000). Our results showed that the aphid nymphs with reduced expression of *Apirp5* presented an increase of soluble carbohydrate and protein contents of whole body. Compared with dsgfp controls, after dsApirp5 treatments, these changing trends of soluble carbohydrate and protein contents in wingless nymphs were similar to that in winged nymphs. This suggested that the changing of nutrient metabolism regulated by *Apirp5* was likely to meet the demand of dispersal of *A. pisum*. Previous studies demonstrate that knockdown and knockout of *dilp2* induce the increase of trehalose in whole body of *Drosophila* (Broughton et al., 2008; Grönke et al., 2010). This implied that the function of *irp* or *ilp* on regulation of soluble carbohydrates was likely to be conserved in insects. Since a swelling mesothorax was not observed after dsApirp5 injection, it was not clear whether an increase of protein content caused by *Apirp5* down-regulation was connected with flight muscle construction. Future histological evidence in the mesothorax would explain whether increased proteins were used to construct flight muscles or not.

The dsRNA treated nymphs showed lower body weight, lower soluble carbohydrate, and higher glycogen than untreated nymphs (Figures 3A,D and Figures 4A,B,D,E). Because of the soft body of aphids, a stab wound made by the capillary resulted in the loss of hemolymph (Altincicek et al., 2008), as suggested by body weight loss. Therefore, it may be reasonable that content of soluble carbohydrate per body weight is decreased while that of glycogen increased, because the main soluble carbohydrate is trehalose in hemolymph and glycogen is contained in the non-hemolymph tissues including fat boy and muscle. The protein concentration, which was similar between injected and non-injected animals (Figures 4C,F), is probably similar in the hemolymph and other tissues.

Although dsApirp5 treatments affected body weights, embryo sizes and body composition, the decrease of *Apirp5* expression did not promote the development of wing apparatus in the nymphs. There were some possible explanations. First, the expression of *Apirp5* was associated with the development of embryos in the ovaries of aphid nymphs during wing differentiation, but *Apirp5* probably did not affect the formation of wing apparatus. The second explanation could be the time point of microinjection. The process of wing differentiation starts in the second instar (Ogawa and Miura, 2013), but it was difficult to inject dsRNA into the first instar nymphs (up to 100% mortality within 48 h in our preliminary analysis). The effects of redundancy among *irp*s on lifespan are found in *Drosophila* (Broughton et al., 2008) and thus may be another reason that only changing *Apirp5* expression levels could not affect wing morph of aphid nymphs, because *Apirp1* in head and *Apirp4, 6*, and *7* in thorax showed significant differences between the two wing morphs. Lastly, low RNAi efficiency has been observed in aphids (Jaubert-Possamai et al., 2007; Christiaens et al., 2014). Our RNAi test also showed the similar result, although we used high concentrations of dsRNA for treatments. Future research will focus on analyzing the compensation and redundancy of *Apirp*s using more efficient gene knockdown methods. Based on existing results, we could not rule out a role for *Apirp5* in wing development.

Other genes in the IS pathway are involved in the regulation of traits associated with trade-offs in other insects. There is only one insulin receptor in Diptera (Fernandez et al., 1995; Ruan et al., 1995), but two insulin receptor homologs are identified in Coleoptera, Hymenoptera, and Hemiptera (Antonova et al., 2012). That raised a question of whether the two insulin receptors had different functions or redundant functions. Although two insulin receptors have been proven to regulate wing polyphenism in the brown planthopper (Xu et al., 2015), in our study, *Apinr1* only showed a significant difference in expression in the midgut between wingless and winged nymphs; therefore, it was hard to establish a link between *Apinr*s and wing polyphenism. *Amirs* RNAi tests on *A. mellifer* have shown a strong link between the IRS and caste fate (Mutti et al., 2011; Wolschin et al., 2011). We found that there were no significant differences in expression levels of *Apirs* in any of the tissues between winged and wingless nymphs in pea aphid. This suggested that *Apirs* might not be the main factor in the IS pathway for regulation of wing differentiation. For *Apide*s, we found that expression of *Apide*1 was higher in winged nymphs than in wingless. There is no evidence that IDEs degrade insect ILPs (Antonova et al., 2012), but knockout of *dide* increases the content of trehalose in *Drosophila* (Tsuda et al., 2010). The connection between sugar metabolism in pea aphid and *Apide*s is worth pursuing in future study.

In this study, we tried to investigate the relationship between the IS pathway and wing polyphenism of pea aphid in the nymphal phase by comparing gene expression levels in the IS pathway and RNAi for one *Apirp*. Our results indicated that *Apirp5* played an important role in regulation of metabolism and development of embryos during wing differentiation in pea aphid. These results gave further recognition on the roles of IS pathway on phenotypic plasticity in aphids.

## Author contributions

SG designed and conducted this study, collected data, analyzed the data, and wrote the paper; MZ helped in all phrases of this study; Prof. TL designed and supervised the whole study, and wrote the paper.

### Conflict of interest statement

The authors declare that the research was conducted in the absence of any commercial or financial relationships that could be construed as a potential conflict of interest.

## Abbreviations

ILP: insulin-like peptide
IRP: insulin-related peptide
InR: insulin receptor
IRS: insulin receptor substrate
IDE: insulin degrading enzyme
HD: high density
LD: low density
IS: insulin signaling
*ilp*s: insulin-like peptide genes
*irp*s: insulin-related peptide genes
*inr*s: insulin receptor genes
*irs*: insulin receptor substrate gene
*ide*s: insulin degrading enzyme genes.
