# Supplementary material for: Insulin-Related Peptide 5 is Involved in Regulating Embryo Development and Biochemical Composition in Pea Aphid with Wing Polyphenism
Source: Front Physiol. 2016 Feb 9;7:31. doi: 10.3389/fphys.2016.00031 (PMC4746287; doi:10.3389/fphys.2016.00031)
Supplement: Supplementary file 1 [file DataSheet1.docx]

**Appendix. Supplementary material**


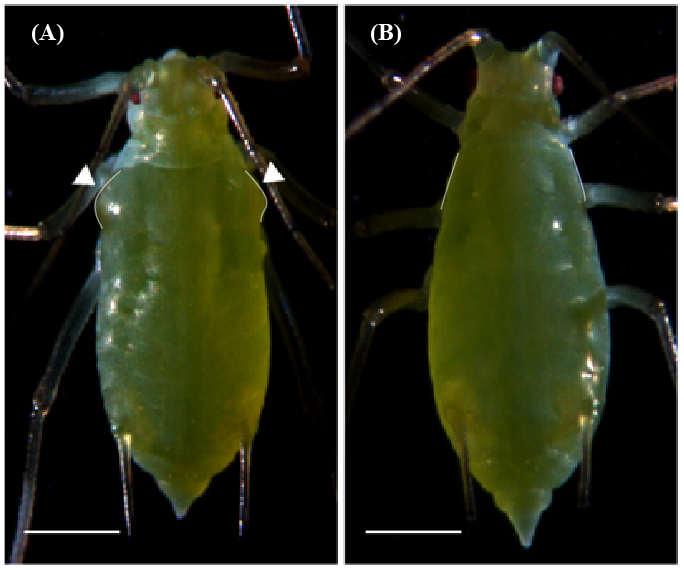


Figure S1. The third instar winged (A) and wingless (B) pea aphid nymphs. White arrow heads show the wing primordia on winged nymphs. White lines outline the structure of the mesothorax of nymphs. The scale bars are 500 μm.


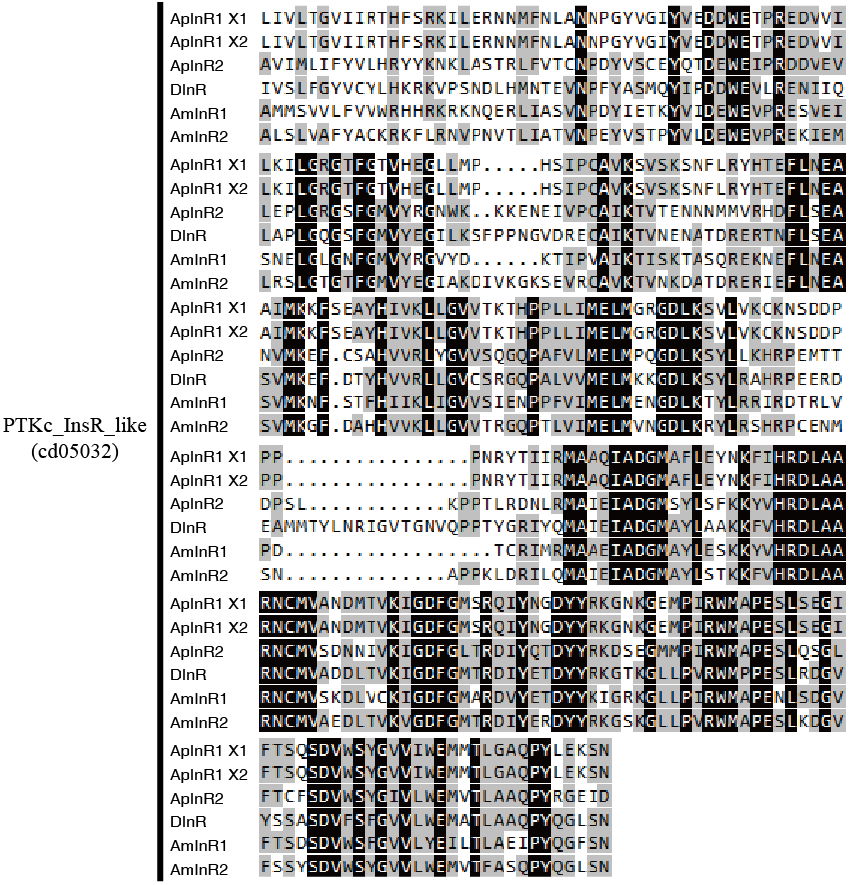


Figure S2. Multiple sequence alignments of conserved domain (PTKc_InsR_like, cd05032) of deduced *Acyrthosiphon pisum* insulin receptors (ApInRs) with other insects’ InRs retrieved from the GenBank database. Identical conserved amino acids identical to *A. pisum* are shown in black, and those with more than 50% homology are shown in gray. ApInR1, *A. pisum*, XP_001942660.2; ApInR2, *A. pisum*, XP_008185917.1; dInR, *Drosophila melanogaster*, AAC47458.1; AmInR1, *Apis mellifera*, XP_394771.5; AmInR2, *A. mellifera*, DAA34971.1.


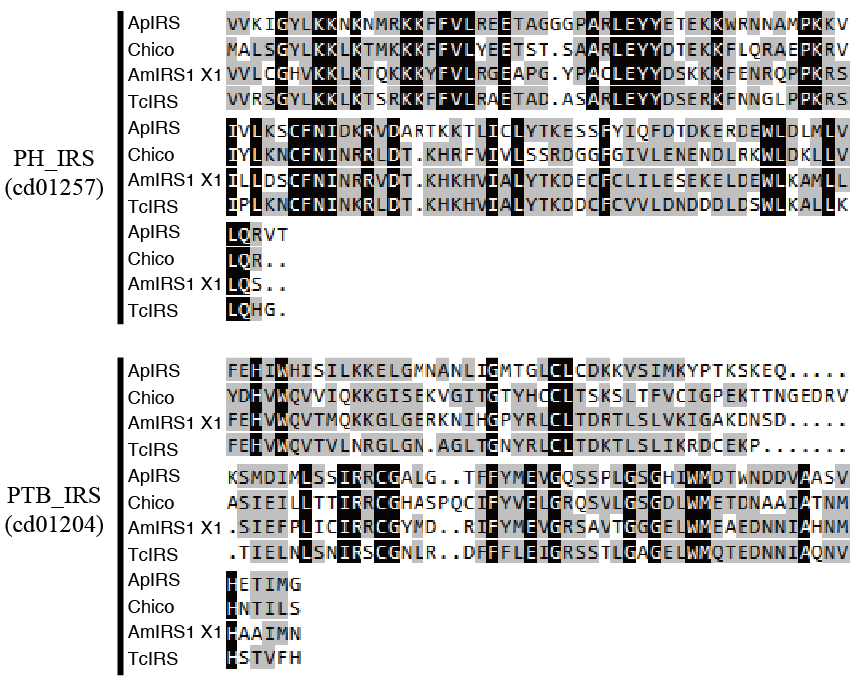


Figure S3. Multiple sequence alignments of conserved domains (PH_IRS, cd01257 and PTB_IRS, cd01204) of deduced *Acyrthosiphon pisum* insulin receptor substrate (ApIRS) with other insects IRS domain sequences retrieved from the GenBank database. Identical conserved amino acids are shown in black and those with more than 50% homology in gray. ApIRS, *A. pisum*, XP_003242430.1; Chico, *Drosophila melanogaster*, ACY01744.1; AmIRS1X1, *Apis mellifera*, XP_006565104.1; TcIRS, *Tribolium castaneum*, XP_008196596.1.


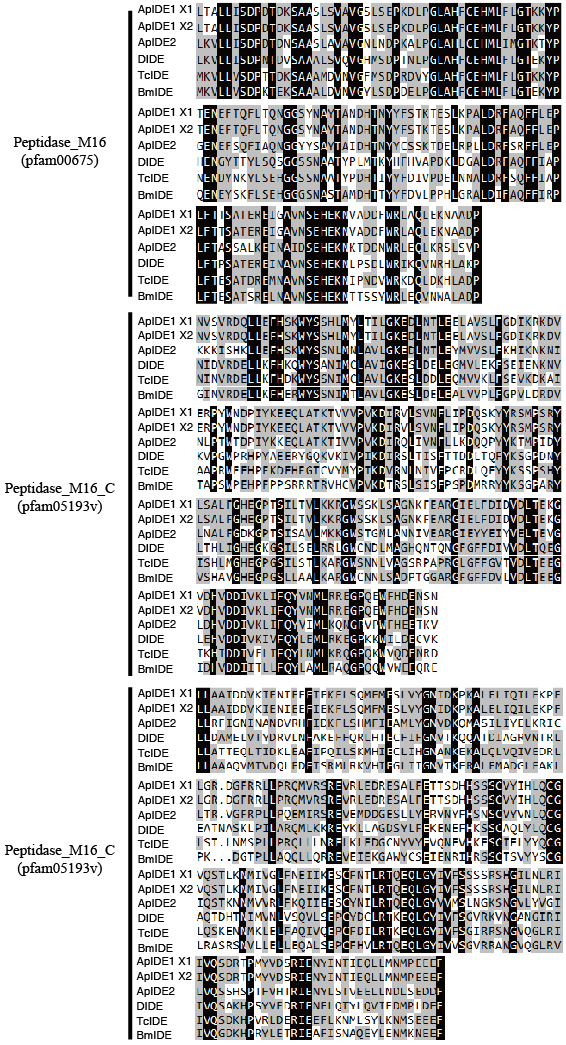


Figure S4. Multiple sequence alignment of conserved domains (Peptidase_M16, pfam00675; Peptidease_M16_C, pfam05193v and Peptidase_M16_C, pfam05193v) of deduced *Acyrthosiphon pisum* insulin degrading-enzymes (ApIDEs) with other insects’ IDE retrieved from the GenBank database. Identical conserved amino acids are shown in black and those with more than 50% homology in gray. ApIDE1, *A. pisum*, XP_001944731.2; ApIDE2, *A. pisum*, XP_001942888.2; dIDE, *Drosophila melanogaster*, AAA28439.1; TcIDE, *Tribolium castaneum* XP_971897.2; BmIDE, *Bombyx mori*, XP_004926345.1.


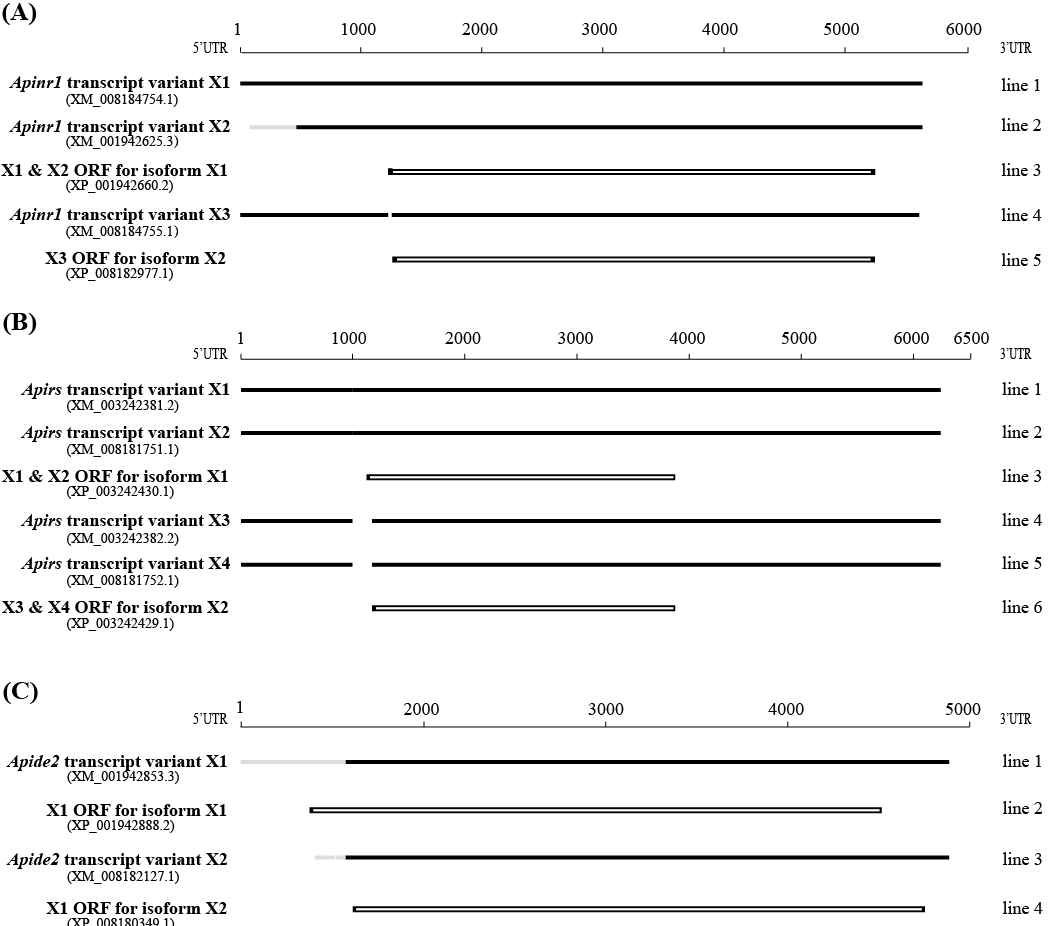


Figure S5. Comparison of *Apinr1*, *Apirs* and *Apide2* mRNA sequences retrieved from NCBL. (A) Three *Apinr1* transcript were found: X1 (XM_008184754.1, line 1), X2 (XM_001942625.3, line 2) and X3 (XM_008184755.1, line 4). X1 and X2 share the same partial ORF of ApInR1 isoform X1 (XP_003242430.1, line 3), and X3 (XM_008187695.1, line 4) has another partial ORF of ApInR1 isoform X2 (XP_008185917.1, line 5). (B) Four transcripts of *Apirs* were retrieved from NCBI. *Apirs* transcript variant X1 (XM_003242381.2) and X2 (XM_008181751.1) share the same ORF of ApIRS isoform X1 (XP_003242430.1), and ApIRS isoform X2 (XP_008182977.1) is translated from *Apirs* transcript variant X3 (XM_003242382.2) and X4 (XM_008181752.1). (C) *Apide2* transcript variant X1 (XM_001942853.3) and X2 (XM_008182127.1) are translated into ApIDE2 isoform X1 (XP_001942888.2) and X2 (XP_008180349.1) respectively. Black line shows the sequences sharing the same parts. Gray line shows the difference between two sequences. Boxes refer to ORF of transcripts.


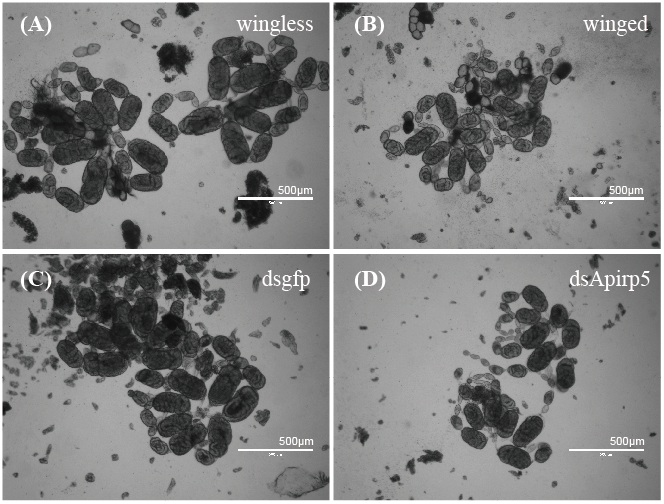


Figure S6. Images of embryos in the ovaries in wingless (A) and winged (B) third instar pea aphid nymphs, and in dsgfp (C) and dsApirp5 (D) injected wingless nymphs. The scale bars are 500 μm.
